# Inferring Seasonal Modulation of Early SARS-CoV-2 Transmissibility from Cross-Country Environmental and Population-Level Predictors

**DOI:** 10.3390/pathogens15090879

**Published:** 2026-08-22

**Authors:** Ognjen Milicevic, Magdalena Djordjevic, Igor Salom, Marko Djordjevic

**Affiliations:** 1 Institute for Medical Statistics and Informatics, Faculty of Medicine, University of Belgrade, 15 Dr Subotica Street, 11000 Belgrade, Serbia; ognjen.milicevic@med.bg.ac.rs; 2 Institute of Physics Belgrade, National Institute of the Republic of Serbia, University of Belgrade, Pregrevica 118, 11080 Belgrade, Serbia; magdalena.djordjevic@ipb.ac.rs (M.D.); igor.salom@ipb.ac.rs (I.S.); 3 Quantitative Biology Group, Faculty of Biology, University of Belgrade, Studentski trg 16, 11000 Belgrade, Serbia

**Keywords:** SARS-CoV-2, COVID-19, seasonal modulation, early-pandemic transmissibility, basic reproduction number, environmental epidemiology, ultraviolet radiation, machine learning, epidemic modeling

## Abstract

Seasonal variation in SARS-CoV-2 transmissibility is difficult to estimate directly from year-round epidemic data because interventions, behavior, reporting, immunity, and viral evolution change concurrently. We therefore asked whether cross-country differences observed during the initial exponential-growth phase could be used to infer country-specific seasonal modulation. Early-pandemic basic reproduction numbers (R_0_) from 118 countries were linked to 96 harmonized environmental and population-level predictors. Among nine candidate algorithms evaluated across 100 repeated train–test splits, ridge regression using the combined predictor set provided the best balance of predictive accuracy, generalization, and temporal stability. The selected model was then driven by daily climatological covariates to reconstruct annual baseline R_0_(t) profiles. Predicted transmissibility generally peaked during winter in the Northern Hemisphere and approximately six months later in the Southern Hemisphere, whereas equatorial countries showed weaker or multimodal patterns. Seasonal forcing amplitude increased strongly with absolute latitude (r = 0.85; mean 0.064 across 77 temperate countries), and predicted R_0_ peaks aligned more closely with minimum ultraviolet radiation than with minimum temperature. These ecological associations do not establish causality, but they provide country-specific seasonal-forcing parameters for epidemic models and a baseline environmental context for comparing early-pandemic trajectories.

## 1. Introduction

Seasonal forcing is a central feature of many respiratory-virus epidemics [1,2], but its contribution to SARS-CoV-2 transmissibility is difficult to isolate from year-round epidemic data. Intervention-focused models have shown that contact tracing, hospitalization, and reductions in transmission-related parameters can substantially reshape epidemic trajectories [3]. In practice, interventions and behavioral responses changed concurrently with epidemic growth, reporting practices varied across countries and time, and the susceptible population changed as the pandemic progressed. Because these processes changed simultaneously, observed associations between environmental conditions and epidemic trajectories are difficult to interpret causally [4,5,6,7,8]. Structural-equation and time-series approaches have been applied to separate climatic and intervention-related effects [9,10], but such analyses remain dependent on the completeness and comparability of the underlying data.

Studies have reported associations of SARS-CoV-2 spread or growth with temperature, humidity, ultraviolet radiation, latitude, and population density, although the magnitude and interpretation of these effects have varied across settings and pandemic phases [4,11,12,13,14,15,16]. However, attributing year-round changes in transmissibility specifically to seasonality remains difficult because the putative seasonal signal is entangled with time-varying epidemiological and societal processes. Consequently, country-specific seasonal-forcing parameters suitable for epidemic models remain difficult to obtain from observational pandemic trajectories alone.

Focusing on the initial exponential-growth phase offers one way to reduce these complications. Early transmission studies used this phase to characterize SARS-CoV-2 growth and estimate transmissibility during the initial spread of the virus [17,18,19]. Approaches based on the initial incidence-growth phase have also yielded comparable country-level R_0_ estimates and related them to environmental and sociodemographic conditions [20,21]. Cross-country variation during this phase therefore provides a comparative setting in which environmental and population-level differences can be related to baseline transmissibility. Here, we use this cross-sectional variation to infer how seasonally varying covariates would modulate baseline transmissibility across an annual cycle.

The resulting parameters are intended for infectious-disease modelers who require country-specific seasonal forcing functions, epidemiologists comparing epidemic trajectories across regions and seasons, and researchers studying environmental determinants of respiratory-virus transmission. They may also provide a more appropriate baseline for retrospective interpretation of public-health interventions.

The purpose of this study was to determine whether early cross-country variation in SARS-CoV-2 transmissibility contains sufficient information to reconstruct country-specific seasonal modulation. The analysis had four aims: (1) to develop predictive models linking early-pandemic R_0_ to harmonized country-level environmental and population profiles across 118 countries (the predictor matrix covered five domains—meteorological conditions, sociodemographic and economic characteristics, population-health indicators, air-pollution exposure, and social connectedness); (2) to combine the fitted dependence of R_0_ on seasonal variables with their annual variation to reconstruct country-specific baseline R_0_(t) profiles and summarize them using the annual baseline level of R_0_, seasonal forcing amplitude A_SF_, and the timing of peak transmissibility t_peak_; (3) to examine how the annual baseline R_0_ and A_SF_ vary with absolute latitude and whether t_peak_ aligns more closely with the annual minimum of ultraviolet radiation or that of temperature; and (4) to use Bayesian inference in selected countries with a clearly identifiable single annual mode to characterize uncertainty and posterior dependence in the inferred seasonal parameters.

This cross-sectional-to-seasonal strategy is designed to infer seasonal R_0_ forcing without fitting transmissibility directly throughout the year. By separating baseline environmental and population-level variation from later time-varying epidemic processes, it provides a practical framework for deriving country-specific seasonal inputs for epidemic models and for interpreting early-pandemic trajectories in their environmental context.

## 2. Materials and Methods

### 2.1. Study Design and Computational Workflow

**Figure 1:**
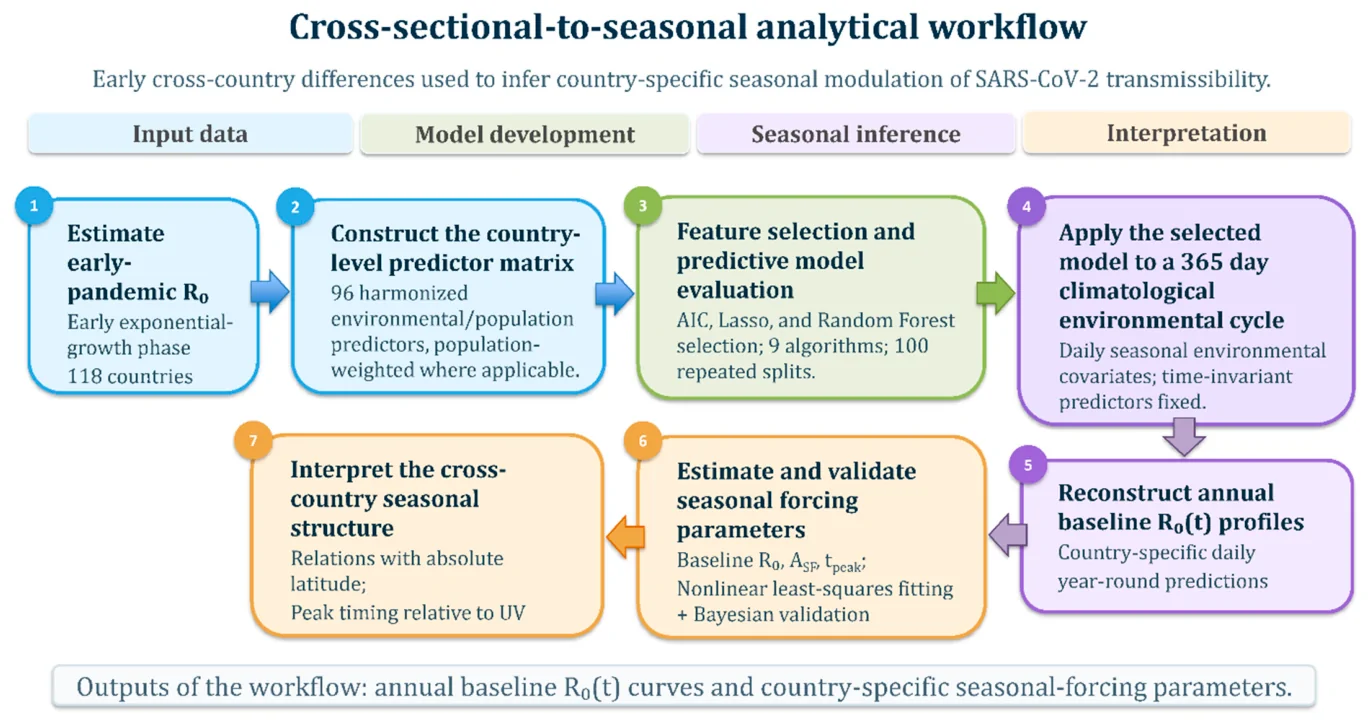
Cross-sectional-to-seasonal analytical workflow. The study begins with estimation of early-pandemic basic reproduction numbers (R_0_) in 118 countries and construction of a harmonized matrix of environmental and population-level predictors. Complementary feature-selection procedures and repeated model validation are then used to identify the final predictive model. The selected model is applied to daily seasonal covariates to reconstruct country-specific annual baseline R_0_(t) profiles, from which seasonal parameters—median R_0_, seasonal forcing amplitude (A_SF_), and timing of peak transmissibility (t_peak_)—are estimated and interpreted epidemiologically. This workflow summarizes the cross-sectional-to-seasonal strategy used to infer baseline seasonal modulation of SARS-CoV-2 transmissibility.

This ecological cross-country study used differences in early-pandemic transmissibility to infer country-specific seasonal modulation of baseline SARS-CoV-2 transmissibility. The overall computational workflow is summarized in Figure 1. First, R_0_ was estimated for 118 countries from the initial, uncontrolled exponential-growth phase, and the resulting values were linked to a harmonized country-level matrix of 96 environmental and population-level predictors. Complementary feature-selection methods and nine candidate algorithms were then evaluated across 100 repeated train–test splits, and the final model was selected based on predictive accuracy, generalization, and temporal stability. The selected model was subsequently applied to daily seasonally varying environmental covariates, with time-invariant predictors held constant, to reconstruct country-specific annual baseline R_0_(t) profiles. Finally, seasonal-forcing parameters were estimated across countries and examined in relation to absolute latitude and the annual minima of ultraviolet radiation and temperature. Complementary Bayesian fits in three selected countries whose reconstructed R_0_(t) profiles each had a clearly identifiable single annual mode were used to characterize posterior uncertainty and parameter dependence. The analysis is ecological and concerns population-level transmissibility rather than within-host viral expression or individual clinical outcomes.

### 2.2. Estimation of Early-Pandemic R_0_

The response variable for the subsequent cross-country analysis was an estimate of early-pandemic transmissibility for each of 118 countries. We inferred this quantity from the initial approximately exponential-growth phase [22,23] using a linearized SPEIRD (Susceptible–Protected–Exposed–Infected–Recovered–Detected) framework [21,24]. In this framework, *D(t)* denotes the cumulative number of detected cases. During the selected early phase, almost the entire population remains susceptible (*S*/*N* ≈ 1), while protection-related transitions and susceptible depletion can be neglected to first approximation. The dynamics relevant to epidemic growth therefore reduce to the Exposed (*E*) and Infected (*I*) compartments:

$$\frac{dE}{dt}\;=\;\beta I\;-\;\sigma E,\;\frac{dI}{dt}\;=\;\sigma E\;-\;\gamma I.$$

where *β* is the transmission rate, *σ* is the rate at which exposed individuals become infectious, and *γ* is the removal rate from the infectious compartment. The growing solution of this linearized system is governed by its dominant positive eigenvalue, *λ_+_*. After the initial transient, and provided that the case-detection process remains approximately stable within the short fitting interval, the cumulative detected-case series follows the same dominant exponential rate:

$$D(t)\;\propto \;e^{\lambda_{+}t},\;\ln\;D(t)\;=\;a\;+\;\lambda_{+}t.$$

where *t* is measured in days and *a* is the fitted intercept. For each country, *λ_+_* was estimated as the slope of a linear fit of ln *D(t)* against time over the country-specific interval exhibiting an approximately linear trajectory on a log-linear plot. The earliest low-count observations were excluded because they were particularly sensitive to stochastic fluctuations, and the fitting interval ended before the visible transition to subexponential growth. The country-specific start and end dates and the resulting slopes are recorded in the publicly available analysis dataset cited in the Data Availability Statement.

The dominant eigenvalue satisfies ${\lambda_{+}}^{2}\;+\;(\sigma \;+\;\gamma )\lambda_{+}\;+\;\sigma (\gamma \;-\;\beta )\;=\;0$. Consequently, the reproduction number under the unprotected early-phase conditions was calculated as

$$R_{0,free}=\frac{\beta }{\gamma }=1+\frac{{\lambda_{+}}^{2}+(\sigma +\gamma )\lambda_{+}}{\sigma \gamma }.$$

Here, *R*_0*,free*_ denotes the basic reproduction number for a fully susceptible population in the absence of effective protection or control. For concision, *R*_0_ ≡ *R*_0,*free*_ throughout the remainder of this article. This quantity should not be confused with the time-dependent effective reproduction number, *R_e_(t)*, which changes in response to interventions and changes in population susceptibility.

For the conversion from *λ_+_* to *R*_0_, the latency-transition and infectious-removal rates were fixed at *σ* = 0.2 day^−1^, corresponding to a mean latency period of 5 days, and *γ* = 0.4 day^−1^, corresponding to a mean infectious period of 2.5 days [25,26,27]. The same parameter values were applied to all countries to ensure a consistent transformation of the estimated growth rates.

Restricting estimation to the initial log-linear interval reduces confounding because susceptible depletion, vaccination, accumulated immunity, later viral evolution, and the major effects of mitigation measures were less likely to have substantially reshaped the observed epidemic trajectory. It does not, however, eliminate all effects of interventions and reporting. Formal control measures or voluntary behavioral changes may already have influenced transmission during part of the fitting interval, while case ascertainment and reporting practices differed among countries. If ascertainment remains approximately stable within a fitting interval, it primarily changes the intercept of ln *D(t)* rather than its slope; changes in testing or reporting during the interval can nevertheless bias *λ_+_* and therefore *R*_0_. The resulting estimates are thus interpreted as standardized approximations of early, minimally controlled population-level transmissibility rather than as perfectly intervention-free or reporting-independent measurements. These 118 country-level *R*_0_ estimates were used as the response variable in the subsequent predictive analyses.

### 2.3. Predictor Domains and Epidemiological Rationale

To characterize country-level conditions that could contribute to differences in early-pandemic transmissibility, we constructed a harmonized matrix of 96 candidate predictors. This matrix expanded the 42-variable set considered in [21]. Rather than treating these variables as an undifferentiated list, we organized them into five complementary thematic domains: (i) meteorological and ultraviolet conditions; (ii) sociodemographic, economic, and urban-structure characteristics; (iii) population-health indicators; (iv) air-pollution indicators; and (v) social connectedness. Predictors were assigned to domains according to the primary construct they measured rather than the database from which they were obtained. Accordingly, meteorological and ultraviolet variables obtained from WAQI were classified within the meteorological-and-ultraviolet domain, whereas WAQI pollutant measurements were classified within the air-pollution domain. The R_0_ outcome, the variables used to derive it (c, start, and end), and Country Code were retained (as shown in Supplementary Table S1) for documentation but were not treated as predictors or assigned to one of the five predictor domains. During construction of the 96-predictor matrix, variables that did not meet the coverage criterion specified in Section 2.4 were excluded.

#### 2.3.1. Meteorological and Ultraviolet Conditions

Meteorological and ultraviolet variables were included to represent physical and seasonal environmental conditions that may influence viral persistence, respiratory-particle behavior, host susceptibility, and seasonal contact patterns [11,12,13,14,15,16]. Daily meteorological time series were obtained with an automated Python 3.9 script using the NASA POWER project service [28]. For each country, daily measurements from major urban centers were aggregated using population weights and then averaged over the country-specific period used for R_0_ estimation. Urban-center coordinates and population data required for this aggregation were obtained from the GHS STAT UCDB2015MT GLOBE R2019A V1.0 database [29].

Ultraviolet radiation was represented using daily maximum UV-index values obtained from the OpenUV service [30]. For each country, these values were retrieved at the coordinates of its largest urban center over the period corresponding to R_0_ estimation. UV radiation was considered because it may affect transmission directly through the inactivation of enveloped viruses on surfaces and in aerosols and indirectly through UV-B-dependent cutaneous vitamin D synthesis and respiratory immune function [31,32,33,34].

The temperature block comprised eight strongly intercorrelated variables: T2M (mean air temperature at 2 m), T2M_MAX, T2M_MIN, T10M, T10M_MAX, T10M_MIN, TS (skin temperature), and an independent temperature estimate from WAQI. To provide a reduced temperature representation for the subsequent feature-selection analysis, we applied principal component analysis (PCA) to this subset [35]. The first two principal components, Tpc1 and Tpc2, together explained 98.2% of the total variance.

#### 2.3.2. Sociodemographic, Economic, and Urban-Structure Characteristics

Sociodemographic, economic, and urban-structure characteristics were included to represent differences in population concentration and mixing, demographic composition, urban form, and the broader socioeconomic context in which early epidemic spread occurred [8]. City-level urban-structure and economic measures were obtained primarily from the Global Human Settlement Urban Centre Database [29], which provides standardized information for more than 10,000 urban centers worldwide. Representative variables included built-up urban area per capita in 2015 and city-level gross domestic product in 2015. Additional country-level indicators characterized population density, urbanization, age structure, migration, and economic development; their definitions and sources are provided in Supplementary Table S1. The geographic coordinates and populations of urban centers from the same database were also used for population-weighted aggregation of meteorological and environmental measurements.

#### 2.3.3. Population-Health Indicators

Population-health indicators were included as ecological markers of potential host vulnerability, behavioral risk factors, and health-system context. They were treated as country-level contextual covariates rather than as individual-level clinical predictors or causal determinants of R_0_. We compiled 25 indicators from the World Health Organization Global Health Observatory [36]. Representative measures included obesity prevalence and mean body mass index, cholesterol and fasting-glucose levels, tobacco and alcohol use, BCG vaccination coverage, hospital-bed availability, health expenditure, and water, sanitation, and hygiene indicators. The complete set is listed in Supplementary Table S1.

#### 2.3.4. Air-Pollution Indicators

Air-pollution indicators were included to represent environmental exposure conditions that may affect respiratory susceptibility and that have previously been associated with respiratory viral infections and with COVID-19 spread or severity [37,38]. Pollution data were obtained from two complementary sources. First, we used the World Air Quality Index (WAQI) COVID-19 compiled historical database, which contains daily city-level measurements of multiple pollutants for the 500 largest cities worldwide. Because its geographic coverage was uneven and resulted in sparse data for some countries, we complemented the WAQI measurements with country-level emissions from the Emissions Database for Global Atmospheric Research (EDGAR) [39]. For nine EDGAR pollutant species, both total national emissions and population-scaled values were considered, yielding 18 variables. These EDGAR measures characterize national emissions rather than direct individual exposure.

#### 2.3.5. Social Connectedness

Social-connectedness variables were included to represent cross-regional interaction pathways that are not captured by local population density or urban structure alone and that may contribute to the spatial spread of an epidemic [40]. We used Facebook’s country–country Social Connectedness Index (SCI), which quantifies social connectedness based on Facebook friendship links. From this weighted country network, we retained two centrality measures: weighted vertex degree, defined as the sum of all edge weights connected to a country, and flow betweenness centrality [41], which characterizes a country’s role in connecting paths through the network.

### 2.4. Data Harmonization and Preprocessing

Data harmonization was designed to make heterogeneous sources comparable at the country level while preventing test-set information from influencing model evaluation. Predictors originally reported at the national level were retained at that resolution. When a source provided measurements for multiple urban centers within a country, the country-level value was calculated as a population-weighted mean, with the contribution of each urban center proportional to its population. For daily meteorological variables, population-weighted aggregation was first performed for each day, after which the resulting country-level values were averaged over the country-specific interval used for R_0_ estimation. Variables represented by a single predefined location within each country, such as OpenUV measurements at the largest urban center, were retained according to the source-specific procedures described above.

Predictor-coverage screening was performed once during construction of the candidate predictor matrix and did not use the R_0_ outcome. Variables with 100 or more missing values among the 118 countries were removed. Per-variable missingness is reported in Supplementary Table S1. Of the 106 candidate predictors considered before coverage screening, 10 had at least 100 missing values and were excluded; of the remaining 96 candidate predictors, 73 contained at least one missing value and were median-imputed, whereas 23 were complete. For each of the 100 repeated 80/20 train–test splits, all subsequent data-dependent preprocessing operations were fitted using the training subset only. Missing predictor values were replaced by the corresponding median calculated from the training subset. Median imputation was used because it is less sensitive than mean imputation to extreme country-level values and skewed predictor distributions.

After imputation, each predictor was automatically assigned one of the following candidate transformations: identity, square/square root, cube/cube root, logarithmic, or exponential. Transformation selection was based exclusively on the training subset, and the selected transformation was subsequently applied unchanged to the corresponding test-set values. Finally, the transformed predictors were centered and scaled using the mean and standard deviation estimated from the training subset. The same training-derived centering and scaling parameters were applied to the test subset. Thus, the held-out observations did not contribute to the imputation values, transformation choices, or standardization parameters used for model fitting and evaluation [42].

### 2.5. Feature Selection and Predictor-Set Construction

Because the number of candidate predictors was large relative to the number of countries (96 predictors for 118 countries), and because several predictors were strongly correlated, we used complementary feature-selection strategies rather than relying on a single selection criterion. The purpose of this stage was to construct a limited number of well-defined predictor sets for systematic comparison in the subsequent predictive-modeling stage. Feature selection was used to support prediction and predictor-set construction, rather than to establish that the retained variables were independent causal determinants of SARS-CoV-2 transmissibility.

We first evaluated two representations of the predictor matrix. The full (“All Variables”) representation retained all original predictors, including the eight strongly intercorrelated temperature-related variables, thereby preserving information at the level of the individual measurements. In the PCA-reduced representation, these eight variables were replaced by their first two principal components (Tpc1 and Tpc2), which together explained 98.2% of their total variance [35]. This representation reduced redundancy associated with the correlated temperature measurements. Comparing the two representations allowed us to assess whether predictor selection and subsequent predictive performance depended on how the correlated temperature information was represented.

We then applied three complementary selection approaches because they capture different aspects of the predictive structure [43,44,45,46]. Stepwise selection based on the Akaike information criterion (AIC) identifies a parsimonious linear predictor set by balancing model fit against model complexity. Lasso uses an L1 penalty to shrink regression coefficients and produce a sparse linear predictor set, which is useful when many candidate variables compete within a relatively small sample. Random Forest permutation importance evaluates the change in predictive performance when individual predictors are permuted and therefore assesses their contributions within a nonlinear, tree-based model capable of representing interactions. Stepwise AIC was applied to the PCA-reduced representation, whereas Lasso and Random Forest permutation importance were each applied separately to both the full and PCA-reduced representations.

This procedure produced five method-and-representation-specific predictor sets. The SAIC (stepwise Akaike information criterion) set contained the variables retained by stepwise AIC from the PCA-reduced representation. Lasso1 and Lasso2 contained the variables selected by Lasso from the full and PCA-reduced representations, respectively, whereas RF1 and RF2 contained the variables identified by Random Forest permutation importance from the corresponding representations. Finally, the Union set comprised every distinct predictor selected in at least one of these five analyses, yielding a sixth predictor set containing 21 predictors. The Union set was included as an inclusive candidate set that reduced the dependence of subsequent model comparison on any single selection procedure; it should not be interpreted as a consensus set containing only predictors supported by every method. The exact composition of all six predictor sets is reported in Table 1.

### 2.6. Model Training, Validation, and Selection

Candidate algorithms were chosen to span ordinary and robust linear models, dimension-reduced and regularized linear models, kernel-based regression, and tree-based ensemble methods, allowing us to assess whether the relationship between the country-level predictors and early-pandemic R_0_ could be adequately represented by a linear model or required a more flexible nonlinear structure. We evaluated nine algorithms: ordinary least squares (OLS) regression, robust regression (RoR), principal component regression (PCR), ridge regression (RiR), Lasso regression (LR), Elastic Net (EN), Random Forest (RF), support vector regression (SVR), and extreme gradient boosting regression (XGB) [42,47,48,49]. Each algorithm was evaluated on each of the six predictor sets described in Section 2.5, yielding 54 algorithm–predictor-set combinations.

For each repetition, countries were randomly assigned to an 80% training set and a 20% held-out test set. As described in Section 2.4, data-dependent preprocessing steps were estimated using the training data and subsequently applied to the test data without refitting. Hyperparameters were tuned exclusively within the training set using fivefold cross-validation and grid search; the held-out test set was not used during model fitting or hyperparameter selection. For algorithms with multiple tunable hyperparameters, the search was performed simultaneously for EN, RF, and SVR and sequentially for XGB. This training, tuning, and evaluation procedure was repeated 100 times using different random seeds to reduce dependence on any single data partition. Tuned hyperparameters were summarized by their modal values across repetitions and are reported in Supplementary Table S2. The complete procedure required more than 2500 CPU-hours on a dedicated computing cluster.

Predictive performance was quantified for each of the 100 repetitions using standardized mean squared error (sMSE) for both the training and held-out test sets. Within each repetition, R_0_ was centered and scaled using the mean and standard deviation estimated from the training set, and the same transformation was applied to the test observations. Thus, sMSE represents the MSE calculated for the standardized outcome. Accordingly, 1−sMSE provides an R^2^-equivalent interpretation of model performance. For held-out predictions, this quantity is not algebraically identical to conventional test-set R^2^ because the prediction errors are calculated in the test set, whereas the variance used for normalization is estimated from the training set. However, because training and test countries were randomly sampled from the same dataset and performance was summarized by the median across 100 repeated splits, it closely approximates conventional R^2^.

For the seasonal-projection screen, the candidate space was restricted to the Lasso1, RF1, and Union predictor sets used in the daily-covariate analysis, yielding 27 algorithm–predictor-set combinations. Only combinations whose 95% empirical interval for test sMSE, derived from the 100 repeated splits, remained below 1.0 were carried forward to the annual-projection stage. The final model was then selected from these acceptable candidates using three complementary criteria: (i) held-out test performance; (ii) the difference between training and test error; and (iii) temporal stability of the projected daily R_0_(t) profiles across the annual covariate cycle. Temporal stability was assessed qualitatively in the illustrative analysis for Germany by examining whether daily predictions followed a coherent annual trajectory without pronounced season-specific increases in short-term scatter. The final choice therefore reflected the overall balance of predictive accuracy, generalization, and suitability for annual projection rather than solely the lowest point estimate of test error. The selected model was subsequently used for the country-specific annual reconstruction described in Section 2.7.

To provide a complementary coefficient-based summary of the Union predictor set, we fitted an auxiliary ordinary least-squares model after predictor-set construction using the 21 Union predictors and standardized predictor and response variables. The absolute standardized coefficients are reported in Supplementary Table S3. This refit was not used for model selection or annual R_0_(t) reconstruction, which remained based on the selected Ridge–Union model. Because the Union set contains correlated predictors, these coefficients were treated as descriptive associations rather than as independent variable contributions or causal effects.

### 2.7. Reconstruction of Annual Baseline R_0_(t)

Annual baseline R_0_(t) profiles were reconstructed using country-specific 365-day climatological environmental cycles. For each meteorological field, a representative value for each calendar day was obtained by averaging the corresponding daily values over the 10-year period 2010–2019. A multi-year daily climatology, rather than observations from any single calendar year, was used to reduce sensitivity to year-specific weather anomalies and to represent the typical seasonal environmental cycle of each country. The climatological series served only as time-varying environmental inputs; the resulting curves therefore represent model-implied baseline transmissibility under a typical annual environmental cycle and should not be interpreted as observed epidemiological transmissibility in 2020, 2021, or any other specific year. This approach captures the typical seasonal pattern but does not represent interannual variability in seasonal forcing.

Daily meteorological and UV values were obtained from the same sources and harmonized using the same country-level procedures described above. The predictors allowed to vary by day were the seasonally varying variables included in the Lasso1, RF1, and Union sets: minimum air temperature at 2 m (T2M_MIN), wet-bulb temperature at 2 m (T2MWET), maximum UV index (OpenUV_max), wind speed (wind.speed), clear-sky surface shortwave irradiance (CLRSKY_SFC_SW_DWN), and all-sky surface shortwave irradiance (ALLSKY_SFC_SW_DWN). All remaining predictors were held at their country-specific baseline values. Missing values within daily series were linearly interpolated, after which the daily inputs were processed using the fitted preprocessing pipeline. For country c and day t, with 1 January defined as t = 1, the prediction was therefore

$${\hat{R}}_{0,c}(t)=\hat{f}\left[x_{c,seasonal}(t),\;x_{c,fixed}\right],$$

where $\hat{f}$ denotes the fitted regression model, $x_{c,seasonal}(t)$ the daily meteorological and UV inputs, and $x_{c,fixed}$ the time-invariant country-level predictors.

All 12 acceptable algorithm–predictor-set combinations were first applied to Germany as an illustrative temperate Northern Hemisphere example, allowing their seasonal patterns and short-term temporal variability to be compared using a common annual input series. The final model selected according to the criteria described in Section 2.6 was then used to reconstruct annual profiles for all 118 countries. For visual presentation 20 countries were chosen to span the latitudinal range and to represent higher-, intermediate-, and lower-latitude Northern Hemisphere settings, equatorial settings, and the Southern Hemisphere. This illustrative subset was not treated as a separate fitting or validation dataset.

### 2.8. Cross-Country Estimation of Seasonal Parameters

To reduce each reconstructed annual baseline $R_{0}\left(t\right)$ profile to a common set of epidemiologically interpretable parameters, we fitted a single-cycle sinusoidal model to the 365 reconstructed daily values for each of the 118 countries using nonlinear least squares. For country c, the fitted function was

$$R_{0,c}^{fit}\left(t\right)=R_{0,median,c}[1+A_{SF,c}cos\left(\frac{2\pi \left(t-t_{peak,c}\right)}{365}\right)].$$

for t=1,…,365, where $R_{0,median,c}$ was fixed at the median of the 365 reconstructed daily baseline $R_{0}$ values for country c, $A_{SF,c}$ was the fitted dimensionless relative amplitude of seasonal forcing, and $t_{peak,c}$ was the fitted day of year at which the sinusoidal approximation reached its maximum. Thus, $A_{SF,c}$ represents the fractional departure from the annual median baseline at the fitted seasonal maximum and minimum. The amplitude was represented as a non-negative quantity, and $t_{peak,c}$ was interpreted modulo 365 days. Nonlinear optimization was performed from two alternative starting parameter sets to reduce sensitivity to local minima, and the converged solution with the smaller residual sum of squares was retained.

The adequacy of the single-cycle sinusoidal representation was quantified separately for each country using ${GOF}_{c}=1-{RSS}_{c}/{TSS}_{c}$, expressed as

$${GOF}_{c}=1-\frac{\sum_{t=1}^{365}[R_{0,c}\left(t\right)-R_{0,c}^{fit}\left(t\right){]}^{2}}{\sum_{t=1}^{365}[R_{0,c}\left(t\right)-{\bar{R}}_{0,c}{]}^{2}}.$$

where $R_{0,c}\left(t\right)$ denotes the reconstructed daily baseline profile, $R_{0,c}^{fit}\left(t\right)$ is its fitted sinusoidal approximation, and ${\bar{R}}_{0,c}$ is the median of the 365 reconstructed daily values. Values closer to 1 indicate that the annual profile is well represented by a single sinusoidal cycle; GOF > 0.90 was used to identify countries with a close sinusoidal fit. GOF was used as a descriptive measure and was not applied as an exclusion criterion in any of the cross-country analyses. Weak or multimodal annual profiles—particularly in equatorial countries—were therefore retained and reported, although they may not be adequately represented by a single annual harmonic.

Cross-country geographic patterns were assessed by calculating Pearson correlation coefficients between absolute latitude and A_SF_, and between absolute latitude and R_0_ (median), using all 118 countries. Countries with absolute latitude greater than 23.5° were classified as temperate, and the distribution of A_SF_ among the resulting 77 temperate countries was summarized by its mean and standard deviation and compared with a fitted Gaussian distribution. For the descriptive comparison of seasonal phase, the analysis was restricted to the 101 countries with |latitude| > 10°, thereby avoiding interpretation of peak dates for the closest-to-equator profiles, where annual patterns were often weak or multimodal. Within this subset, t_peak_ was compared with the dates on which the corresponding country-level climatological environmental series reached their annual minima: maximum daily UV index (${OpenUV}_{max}$) and minimum air temperature at 2m (T2M_MIN).

Phase agreement was quantified as the shortest circular calendar distance,

$$d_{x,c}=min\left(\left|t_{peak,c}-t_{min,x,c}|,365-|t_{peak,c}-t_{min,x,c}\right|\right).$$

where x∈{UV,T}, so that dates close to one another across the calendar-year boundary were treated as temporally adjacent. Smaller values indicate closer alignment between the fitted $R_{0}$ peak and the corresponding environmental minimum. Because UV radiation and temperature contributed to the environmental inputs used to reconstruct the annual $R_{0}\left(t\right)$ profiles, these phase comparisons were interpreted descriptively and not as independent causal validation. Cross country seasonal parameter analysis was done in MATLAB R2023b (MathWorks).

### 2.9. Bayesian Characterization of Seasonal Parameters in Selected Countries

Following the reconstruction of annual baseline $R_{0}\left(t\right)$ profiles described in Section 2.7 and the frequentist estimation of seasonal parameters across all 118 countries described in Section 2.8, we performed a complementary Bayesian analysis to quantify parameter uncertainty and posterior dependence in three selected countries whose reconstructed $R_{0}\left(t\right)$ profiles had a clearly identifiable single annual mode and were therefore suitable for the assumed single-cycle sinusoidal model. Lithuania (54.7° N) and Serbia (44.8° N) were included as Northern Hemisphere examples at different latitudes, whereas Argentina (34.6° S) was included to examine the expected hemispheric reversal in seasonal phase. This selected-country analysis was illustrative and was not intended to represent the full range of latitudes or annual profile shapes; cross-country inference was based on the analyses of all 118 countries described in Section 2.8.

For each selected country, the 365 daily baseline $R_{0}$ predictions generated by Ridge regression using the Union predictor set were treated as observations from the following sinusoidal model:

$$R_{0,d}^{pred}∼N\left(\mu_{d},\sigma^{2}\right),\;\;\mu_{d}=R_{base}\left[1+A_{SF}cos\left(\frac{2\pi \left(d-t_{peak}\right)}{365}\right)\right],\;\;d=1,\ldots ,365.$$

Here, $R_{0,d}^{pred}$ is the model-predicted baseline reproduction number on day d of the year, $\mu_{d}$ is its expected value under the sinusoidal model, $R_{base}$ is the annual baseline level of the fitted curve, and $A_{SF}$ is the dimensionless relative amplitude of seasonal forcing. The phase parameter $t_{peak}$ denotes the day of year on which the fitted $R_{0}\left(t\right)$ curve reaches its maximum, and σ is the residual standard deviation of the daily predictions around the sinusoidal curve.

The prior distributions were specified as

$$R_{base}∼N\left(R_{0,median},{\left[0.35R_{0,median}\right]}^{2}\right),\;\;A_{SF}∼N\left(0.35,0.05^{2}\right),$$

$$t_{peak}∼N\left(t_{min,T},30^{2}\right),\;\;\sigma ∼LogNormal\left(0,1\right).$$

Here, $R_{0,median}$ is the median of the 365 daily predictions for the corresponding country, as defined in Section 2.8, and $t_{min,T}$ is the country-specific day of minimum annual temperature. The prior for $R_{base}$ was therefore centered on the frequentist annual baseline with a coefficient of variation of 0.35, whereas the prior for $t_{peak}$ allowed a standard deviation of 30 days around the temperature minimum. The informative prior for $A_{SF}$ was based on preliminary seasonal-amplitude estimates, while the lognormal prior on σ provided a broad positive prior for residual variability. For the lognormal distribution, the stated parameters refer to the location and scale on the logarithmic scale.

The model was implemented in R and fitted using Hamiltonian Monte Carlo sampling in Stan [50]. Four independent chains of 2000 iterations were run for each country, with the first 1000 iterations of each chain used for warmup. This yielded a total of 4000 post-warmup posterior draws per country. When the frequentist estimate of $t_{peak}$ occurred before day 60 or after day 300, the daily series was circularly shifted by 183 days before Bayesian fitting to avoid a discontinuity in the phase parameter near the calendar-year boundary. After sampling, the posterior phase estimates were transformed back to the original day-of-year scale.

Posterior distributions were obtained for $R_{base}$, $A_{SF}$, $t_{peak}$, and σ and were summarized using posterior means, credible intervals, and marginal and joint posterior distributions. Convergence was evaluated by inspecting between-chain mixing in the trace plots and the $\hat{R}$ diagnostic, while sampling efficiency and potential pathologies were assessed using effective sample sizes and divergent-transition diagnostics.

## 3. Results

The final analytic dataset included 118 countries and 96 predictors grouped into the five thematic domains defined in Section 2.3. Temperature-related meteorological variables were highly intercorrelated and were summarized by PCA; the first two components retained 98.2% of the variance. We then used exploratory linear diagnostics, feature selection, predictive-model evaluation, year-round R_0_(t) reconstruction, and seasonal-parameter fitting to follow the analytical sequence defined in the Introduction.

### 3.1. Initial Exploration and Outlier Analysis

**Figure 2:**
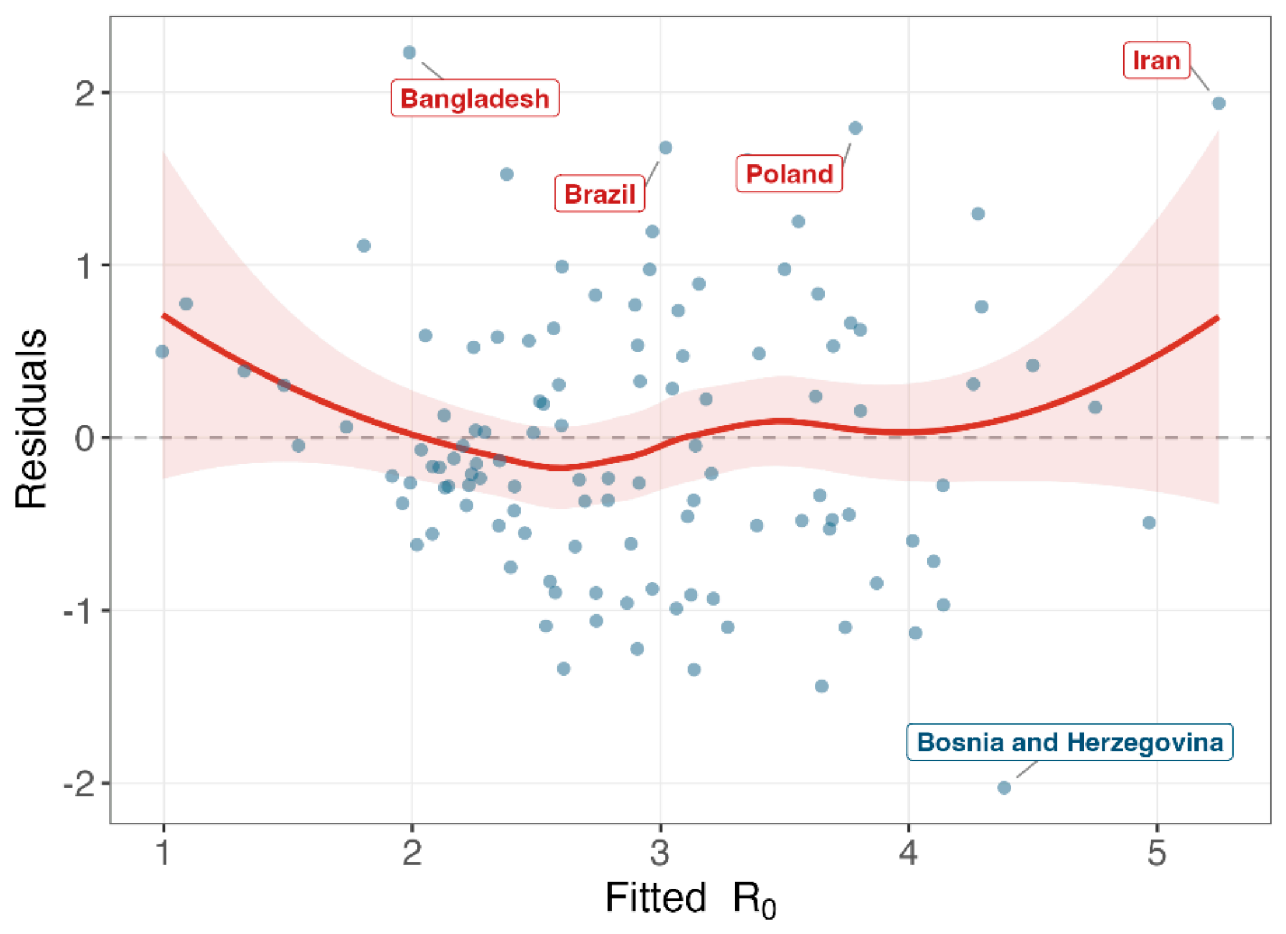
Residuals-versus-fitted plot from the initial ordinary least squares regression on the full predictor set. Bangladesh, Brazil, Poland, and Iran show positive residuals, whereas Bosnia and Herzegovina shows a markedly negative residual. These countries are labeled to highlight the main deviations from the model fit.

**Figure 3:**
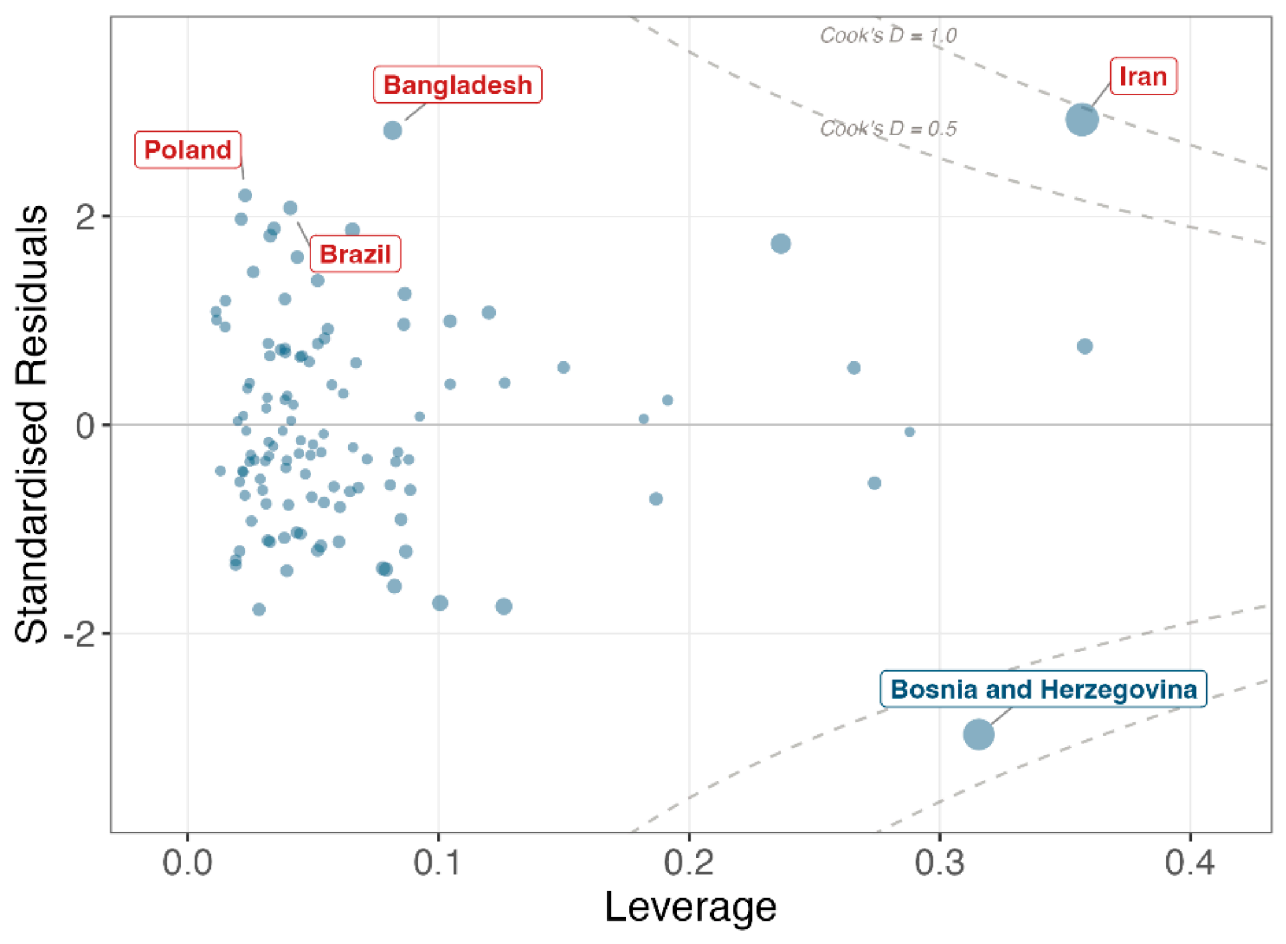
Residuals-versus-leverage plot from the initial ordinary least squares regression. Iran and Bosnia and Herzegovina appear as the most influential observations, while Bangladesh also shows elevated residual magnitude with lower leverage. Dashed curves denote Cook’s distance.

As an exploratory diagnostic, we first fit an ordinary least squares model to the full predictor set. The residuals-versus-fitted plot highlighted several countries with unusually large residuals, most notably Bangladesh, Brazil, Poland, and Iran on the positive side and Bosnia and Herzegovina on the negative side (Figure 2). The residual-leverage plot showed that Iran and Bosnia and Herzegovina were also influential observations, with Bangladesh showing more moderate leverage (Figure 3) [51].

We found no obvious downloading or processing errors, so these cases were retained as likely reflecting genuine cross-country heterogeneity rather than technical artifacts.

### 3.2. Feature Selection

To link early-pandemic R_0_ to country-specific environmental and population-level profiles, we performed feature selection using stepwise AIC, Lasso shrinkage, and Random Forest permutation importance. The resulting predictor sets are shown in Table 1. We also defined a Union set containing all variables selected by at least one method, yielding 21 predictors for final model evaluation.

The validation stage yielded the hyperparameters shown in Table S2. Reported values are modes across the 100 repeated train–test splits with fivefold cross-validation performed within each training set.

### 3.3. Model Evaluation

Predictive performance was evaluated for all nine algorithms on all six predictor sets. Median standardized train and test sMSE values across 100 repeated splits are shown in Table 2.

**Figure 4:**
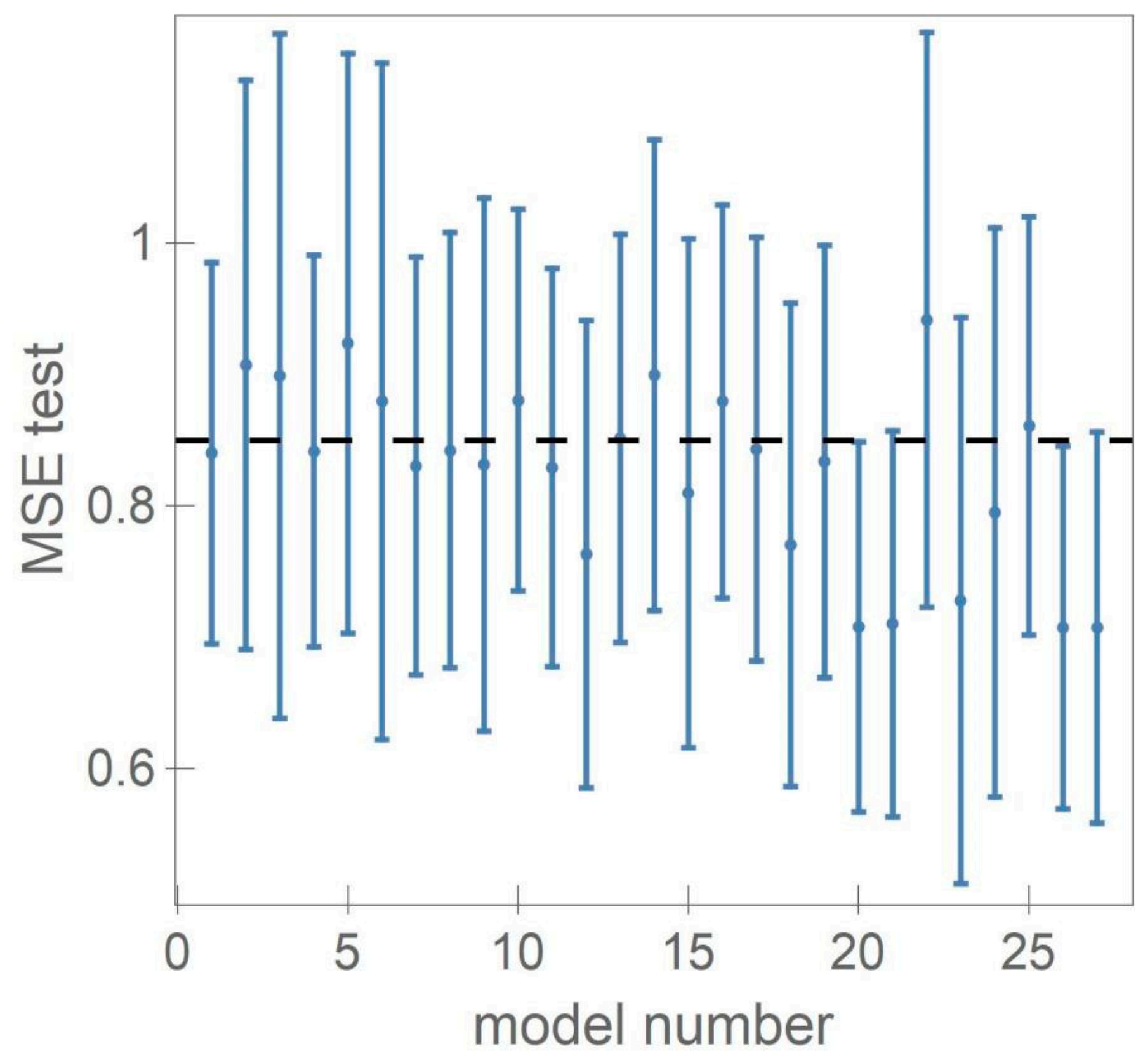
Test sMSE for 27 combinations of algorithm and predictor set (nine algorithms × three predictor sets containing meteorological variables: Lasso1, RF1, and Union). Intervals are derived from 100 repeated random train-test splits. The dashed horizontal line indicates the performance threshold; 12 combinations falling consistently below 1.0 were retained for year-round R_0_ prediction.

For the seasonal-projection stage, we focused on the three predictor sets that contain meteorological variables: Lasso1, RF1, and Union. This yielded 27 algorithm–predictor combinations. Twelve had median test sMSE consistently below 1.0 and were retained as acceptable models for year-round prediction (Figure 4). For coefficient-based interpretability, Supplementary Table S3 reports the absolute standardized coefficients from a post-selection ordinary least-squares refit on the 21-variable Union set. This refit was descriptive and was not used to select the final model or reconstruct annual R_0_(t) profiles.

### 3.4. Annual Baseline R_0_(t) Reconstruction

**Figure 5:**
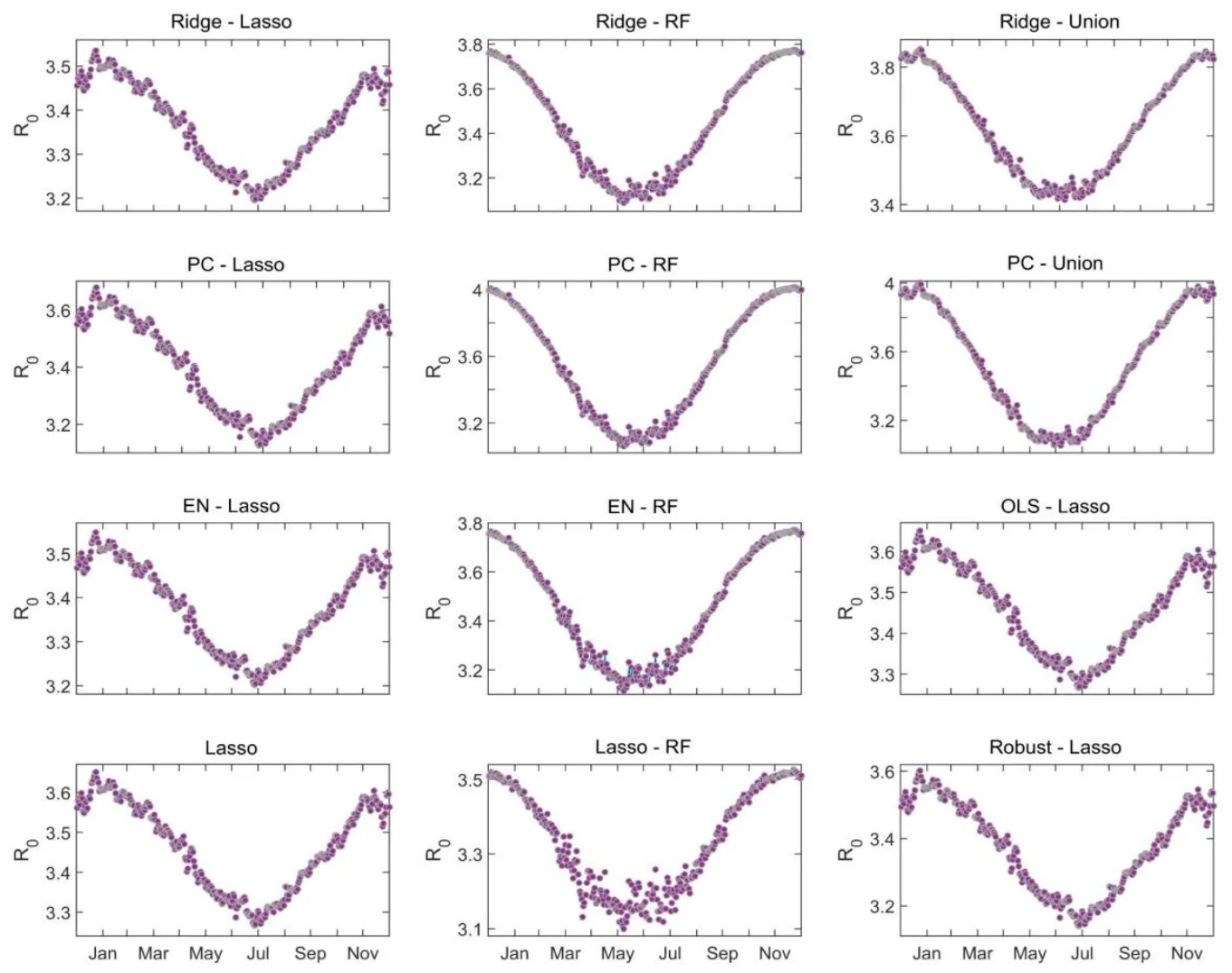
Baseline predicted daily R_0_(t) for Germany under the 365-day climatological environmental cycle constructed from 2010–2019 data, generated by the 12 acceptable algorithm–predictor-set combinations. Each panel identifies the algorithm and predictor set used. Union-based combinations produced the most temporally uniform daily profiles.

Across the 12 acceptable algorithm–predictor-set combinations, the reconstructed annual profiles for Germany showed broadly similar seasonal variation, with lower predicted baseline R_0_ values during spring and summer (Figure 5). Profiles based on Lasso1 showed greater short-term variability during colder periods, whereas those based on RF1 were more variable during warmer periods. Union-based predictions showed the greatest temporal uniformity.

Ridge regression on the Union predictor set was selected as the final model because it combined competitive predictive performance (median standardized train and test sMSE = 0.72 and 0.78, respectively), a small train–test discrepancy, and stable daily projections. Although Random Forest on SAIC and XGBoost on Lasso1 yielded lower median test-sMSE point estimates (0.70 and 0.74, respectively), SAIC contained no seasonally varying predictors, whereas XGBoost on Lasso1 showed a larger train–test discrepancy (0.50 versus 0.74) and did not satisfy the full repeated-split retention criterion. Among the retained combinations, Ridge regression on Union also produced the most temporally uniform daily profile.

**Figure 6:**
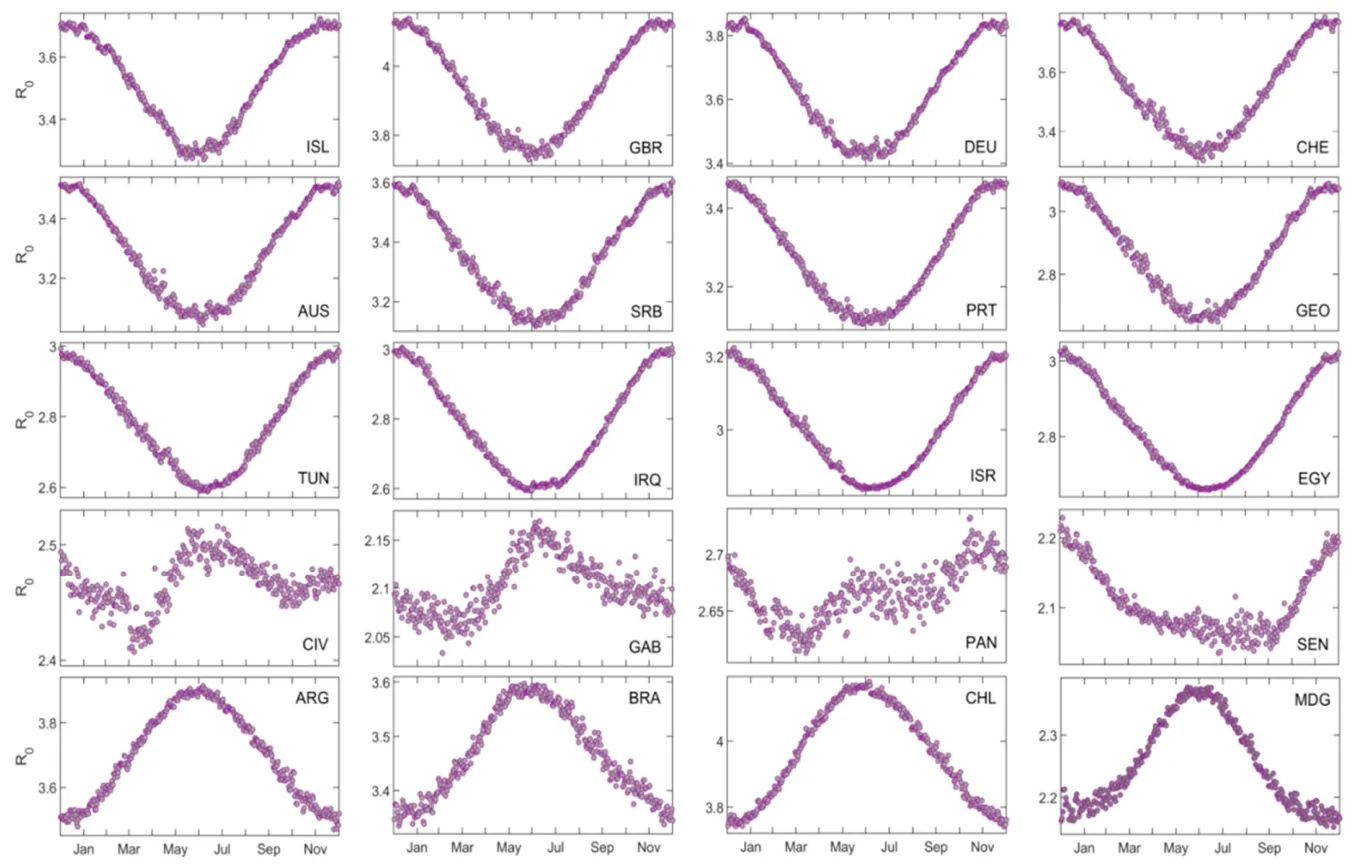
Baseline predicted daily R_0_(t) under country-specific 365-day climatological environmental cycles constructed from 2010–2019 data for 20 illustrative countries spanning a wide range of latitudes, generated by Ridge regression on the Union predictor set. Rows show higher-, intermediate-, and lower-latitude Northern Hemisphere examples, equatorial examples, and Southern Hemisphere examples, respectively.

The selected Ridge–Union model was used to reconstruct annual baseline R_0_(t) profiles for all 118 countries; Figure 6 presents the 20-country illustrative subset. Northern Hemisphere countries generally showed lower predicted R_0_ values during mid-year, Southern Hemisphere countries showed the opposite seasonal pattern, and equatorial countries showed weaker or multimodal variation with smaller annual amplitudes.

### 3.5. Bayesian Seasonal Analysis

To provide an illustrative characterization of parameter uncertainty for profiles with a clear annual mode, we fitted the Bayesian sinusoidal model to three selected countries from both hemispheres. This yielded posterior distributions for R_base_, A_SF_, seasonal phase, and residual variability.

**Figure 7:**
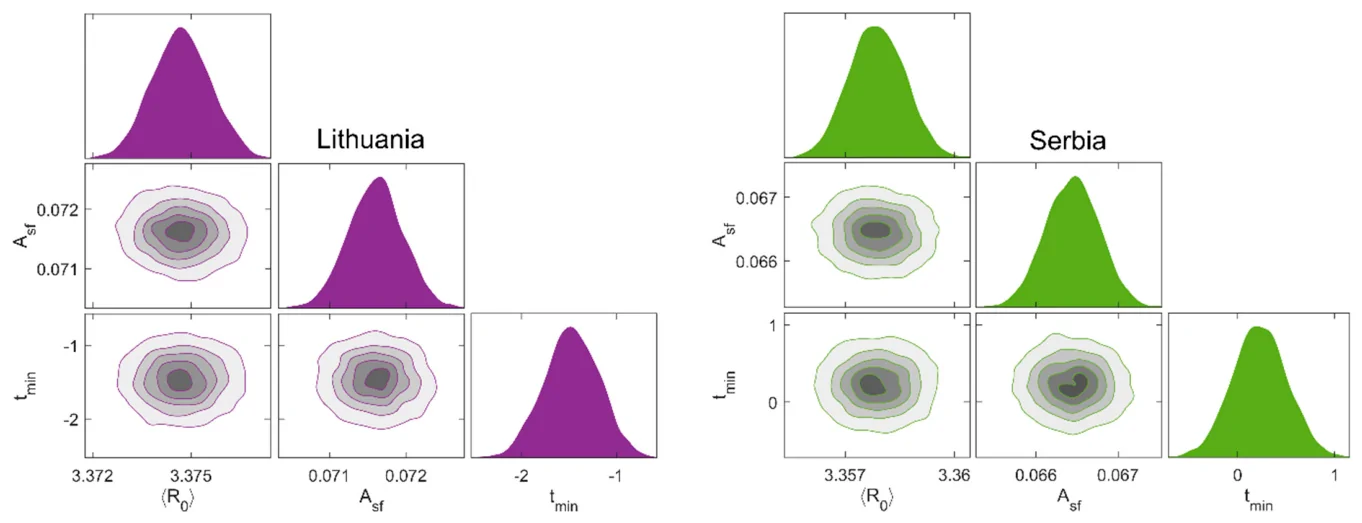
Bayesian seasonal fits for two Northern Hemisphere countries. (**Left**) Posterior corner plot for Lithuania (54.7°N), with posterior mean estimates R_base_ = 3.38 and A_SF_ = 0.072. (**Right**) Posterior corner plot for Serbia (44.8°N), with posterior mean estimates R_base_ = 3.36 and A_SF_ = 0.066. Diagonal panels show marginal posterior distributions; off-diagonal panels show joint posterior distributions. Narrow credible intervals and limited parameter correlations indicate stable parameter identification.

The Northern Hemisphere examples showed similar baseline transmissibility and winter-peaked seasonal structure. Lithuania, which is farther from the equator, showed a slightly larger seasonal amplitude (A_SF_ = 0.072) than Serbia (A_SF_ = 0.066), consistent with stronger seasonal forcing at higher latitude (Figure 7).

**Figure 8:**
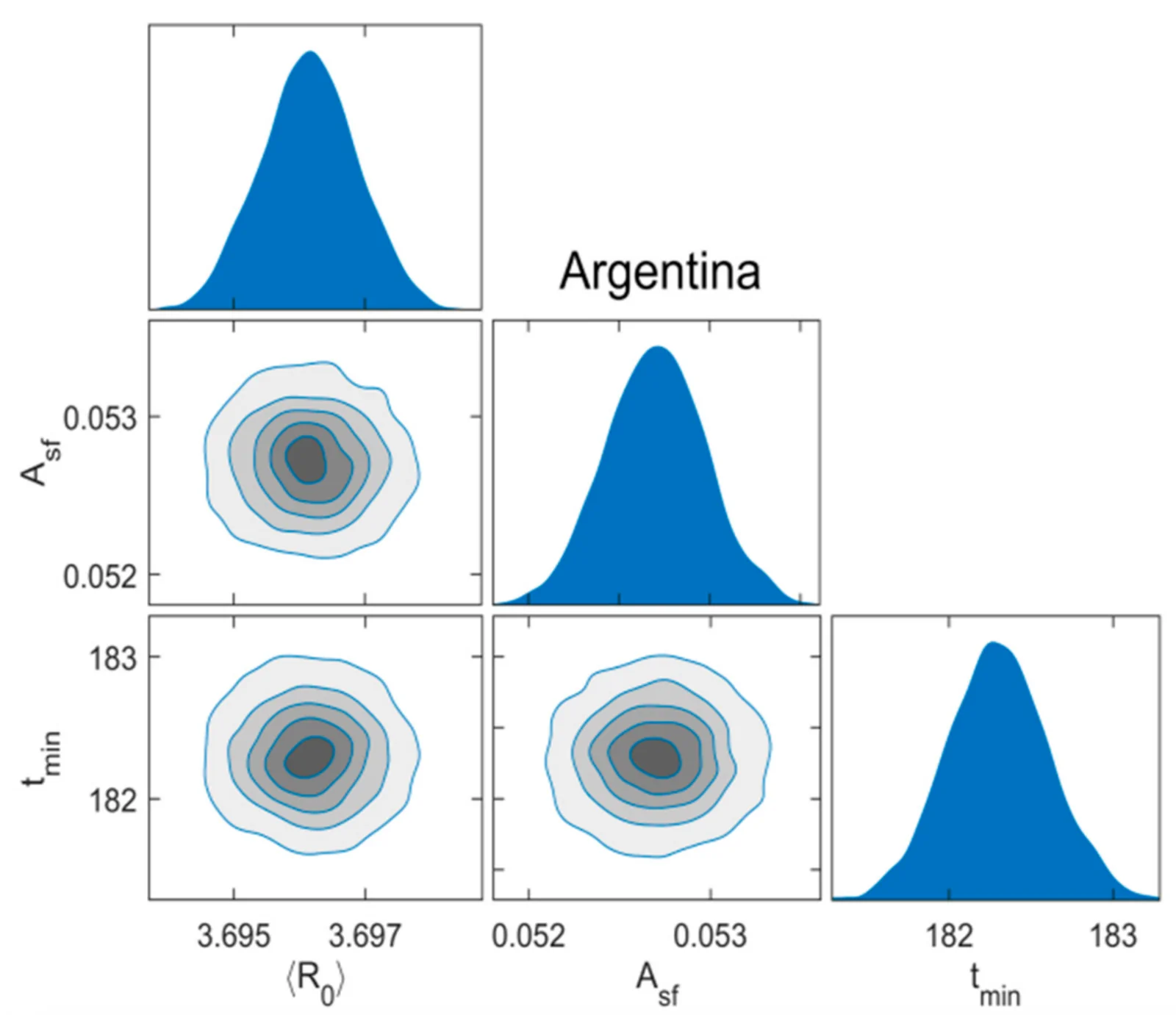
Bayesian posterior corner plot for Argentina (34.6°S). Posterior mean estimates were R_base_ = 3.70 and *A_SF_* = 0.053. The fitted phase is shifted by approximately six months relative to the Northern Hemisphere examples, consistent with hemisphere-dependent seasonal forcing.

Argentina (34.6°S) showed the expected phase reversal relative to the Northern Hemisphere countries. Its posterior mean R_base_ was 3.70 and A_SF_ was 0.053, with the fitted seasonal phase shifted by approximately six months relative to Serbia and Lithuania (Figure 8).

**Figure 9:**
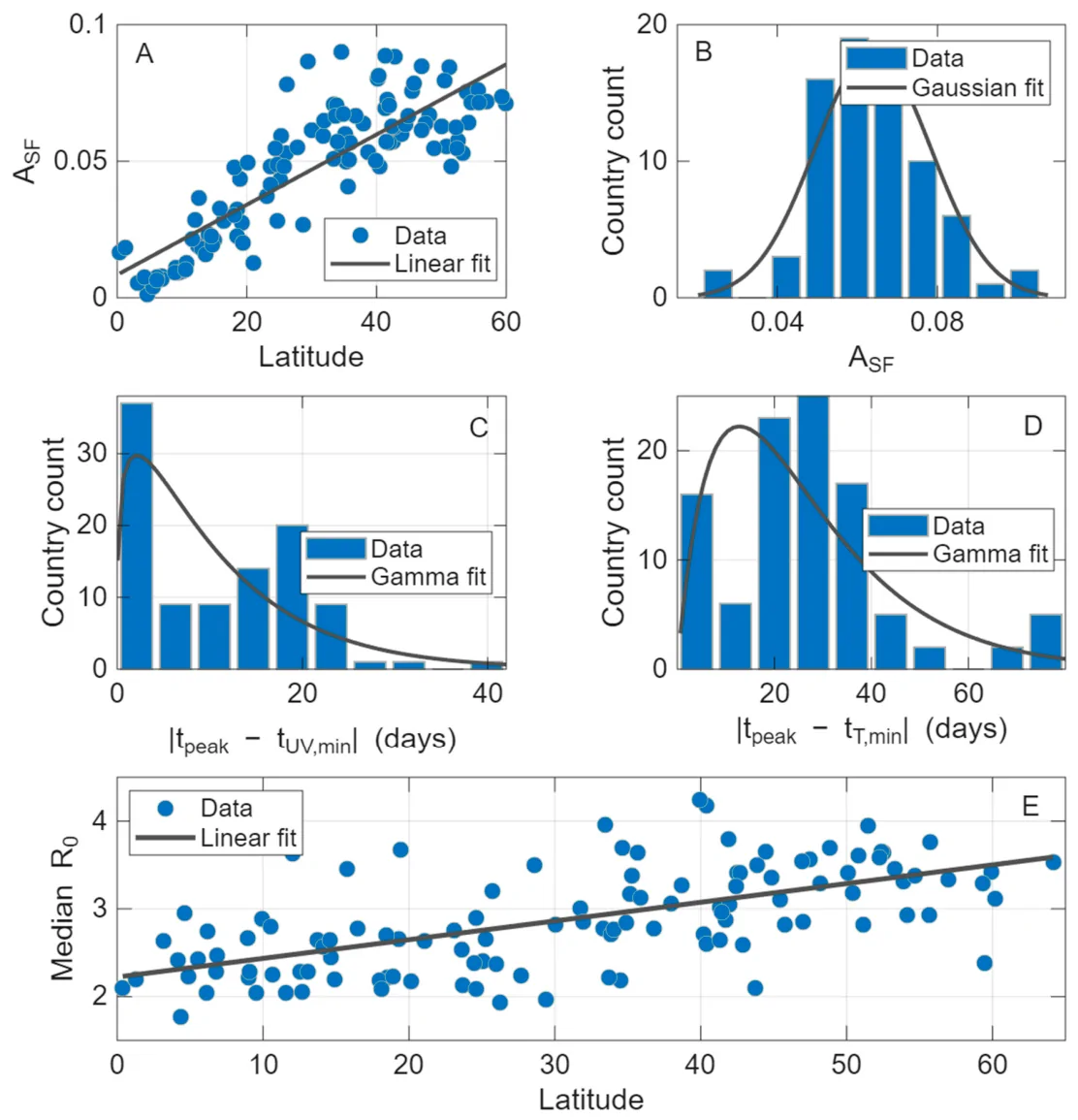
Cross-country analyses of seasonal R_0_ parameters. (**A**) Seasonal forcing amplitude A_SF_ as a function of absolute latitude for all 118 countries, showing a strong positive relationship (r = 0.85). (**B**) Distribution of A_SF_ among the 77 temperate countries (|latitude| > 23.5°), with a Gaussian fit (mean = 0.064, SD = 0.015). (**C**) Shortest circular calendar distance in days between the R_0_ peak and the OpenUV radiation minimum among the 101 countries with |latitude| > 10°. (**D**) Corresponding distance between the R_0_ peak and the minimum temperature (T2M_MIN) among the same 101 countries. (**E**) Median annual R_0_ as a function of absolute latitude for all 118 countries, showing a positive linear trend (r = 0.59).

Together, these Bayesian fits provide a simple quantitative characterization of the magnitude and timing of seasonal forcing in profiles with a clearly identifiable single annual mode. They are illustrative rather than geographically representative; broader geographic inference is based on the 20-country profile comparison in Figure 6 and the frequentist analysis of all countries in Figure 9.

### 3.6. Cross-Country Analysis

After reconstructing country-specific R_0_(t) profiles, we estimated seasonal parameters for all 118 countries using nonlinear least squares. Panel-specific country sets were then used to examine the spatial structure of seasonal forcing and the timing of peak predicted transmissibility (Figure 9).

Seasonal amplitude increased strongly with absolute latitude across all 118 countries (r = 0.85; Figure 9A), indicating stronger seasonal modulation of predicted R_0_ farther from the equator [12,15,16]. Among the 77 temperate countries, A_SF_ was approximately Gaussian with mean 0.064 and SD 0.015 (Figure 9B). Among the 101 countries with |latitude| > 10°, the fitted R_0_ peak aligned more closely with the OpenUV minimum than with the temperature minimum: many peaks fell within 5 days of the UV minimum, whereas the temperature-based distribution was broader (Figure 9C,D). This is consistent with UV radiation as a plausible proximal driver of transmission seasonality, although it does not establish causality [12,15,16]. Median annual R_0_ also increased with absolute latitude across all 118 countries (r = 0.59; Figure 9E). The sinusoidal model achieved good fit (GOF > 0.9) for 73 of 118 countries, with poorer fits concentrated among equatorial countries where the predicted seasonal pattern was weak or multimodal.

## 4. Discussion

The principal finding is that a coherent, hemisphere-dependent seasonal signal can be recovered from cross-country variation in early-pandemic R_0_, even though the training data did not span a complete annual cycle. Seasonal amplitude increased strongly with absolute latitude, and predicted transmissibility peaks aligned more closely with UV minima than with temperature minima. The methodological contribution is the use of an early cross-sectional epidemiological baseline to derive country-specific annual forcing functions without fitting directly to later pandemic trajectories.

The estimated seasonal parameters were consistent with the central expectation of respiratory-virus seasonality. Northern and Southern Hemisphere countries showed approximately inverted annual patterns, and seasonal amplitude increased strongly with absolute latitude. This result is notable because the training data were limited to the early phase of the pandemic, before any country had completed a full seasonal cycle. The emergence of the expected hemisphere-dependent seasonality suggests that the environmental covariates captured seasonally relevant information rather than a temporal artifact [1,2,4,13,14,15,52].

The Bayesian fits provided an illustrative quantification of seasonal variability and parameter uncertainty for three selected profiles with a clearly identifiable single annual mode. Serbia and Lithuania showed similar winter-peaked seasonal structure, with the higher-latitude country showing a somewhat larger seasonal amplitude. Argentina showed the expected approximate six-month phase shift relative to the Northern Hemisphere examples. These selected-country fits were not used to establish geographic generalizability; the evidence for weaker or multimodal low-latitude patterns, hemispheric phase reversal, and increasing seasonal amplitude with absolute latitude comes from the broader reconstructed-profile and all-country frequentist analyses (Figure 6 and Figure 9).

The alignment between fitted R_0_ peaks and UV minima provides a plausible mechanistic clue. UV radiation can inactivate SARS-CoV-2 under simulated sunlight conditions, and UV-B exposure contributes to cutaneous vitamin D synthesis, while vitamin D status has been linked to acute respiratory infection risk [31,32,33,34]. However, this is an ecological predictive analysis, not a causal experiment. The closer alignment with UV minima than with temperature minima could therefore be interpreted as support for UV radiation as a candidate proximal driver of seasonality, not as proof that it is the dominant causal mechanism.

Because several meteorological and ultraviolet predictors in the Union set are strongly correlated, the absolute standardized coefficients from the auxiliary OLS refit (Supplementary Table S3) share explanatory information and should be interpreted as model-conditional associations rather than as independent contributions or causal effects. In particular, the comparatively small coefficient for OpenUV_max does not quantify the importance of UV for the reconstructed seasonal pattern: the closer alignment of t_peak_ with the UV minimum than with the temperature minimum reflects the phase structure of the full Ridge–Union projection and should not be interpreted as evidence of an independent UV effect.

The policy implication is not that environmental factors determine epidemic outcomes, but that they shape the baseline conditions under which interventions operate. Comparisons of national policy effectiveness or outbreak severity can be misleading if they ignore differences in season, latitude, urban structure, health indicators, and social connectedness. Accounting for baseline seasonal forcing may improve retrospective evaluation of interventions and help define more appropriate counterfactual expectations for early epidemic growth.

Several limitations should be noted. First, missing data for several retained predictors were substantial and unlikely to be missing completely at random; missingness is related to reporting infrastructure, economic development, and data governance. Five of the six predictors allowed to vary in the annual reconstruction were complete or nearly complete in the cross-country fitting matrix, whereas wind speed had 54 of 118 values missing; other selected sparsely observed predictors were held fixed during reconstruction and therefore did not directly generate daily variation. Second, country-level aggregation obscures within-country heterogeneity and cannot measure individual exposure directly. This is particularly relevant for air-pollution variables: WAQI station measurements are closer to exposure but sparse, whereas EDGAR provides broad national coverage but measures emissions rather than personal exposure. Third, seasonality was captured through environmental covariates, whereas behavior, mobility, indoor crowding, and school or workplace patterns may add further seasonal structure. This limitation is consistent with machine-learning forecasting work in Japan showing that public mobility can dominate short-term COVID-19 case prediction, while meteorological inputs may still improve predictive accuracy in some regions [53]. Fourth, the sinusoidal model assumes a single annual mode, which is appropriate for many temperate countries but less suitable for equatorial countries with weak or multimodal seasonality. Finally, the Bayesian amplitude prior was centered higher than the posterior estimates; although the likelihood dominated the prior, formal prior-sensitivity analysis would strengthen this component.

Despite these limitations, the study provides a coherent framework for estimating baseline seasonal and population-level forcing separately from later policy-driven changes in epidemic trajectories. The resulting parameters—median R_0_, seasonal forcing amplitude, and timing of peak transmissibility—can be used to incorporate country-specific seasonal forcing into longer-term models of SARS-CoV-2 transmission and to interpret early pandemic trajectories in their environmental context.

## 5. Conclusions

Early SARS-CoV-2 transmissibility varied substantially across countries, and a seasonal component could be inferred from environmental and population-level covariates. Ridge regression on the Union predictor set provided a robust final model for projecting annual R_0_(t) profiles. Cross-country fitting showed that seasonal amplitude increased strongly with absolute latitude, while the timing of peak predicted R_0_ aligned more closely with minimum UV radiation than with minimum temperature. Bayesian fits in three illustrative countries quantified uncertainty in the inferred seasonal parameters and illustrated the hemispheric phase shift for profiles with a clear annual mode. These results provide a practical set of country-specific seasonal-forcing parameters for longer-term epidemic models and support accounting for baseline environmental context when comparing early pandemic trajectories and public-health interventions.

## Figures and Tables

**Table 1:** Six predictor sets obtained using feature-selection methods. Variable definitions are given in Supplementary Table S1.

Model	Prediction Formula (Linear Notation Used Even for Non-Linear Models)
SAIC	R_0_~so2 + Weight + NMVOC_pc + o3 + PPP.per.capita + fast_glucF + obesity_prev
Lasso1	R_0_~T2M_MIN + so2 + CholesterolF + total_alc
Lasso2	R_0_~T2MWET + ALLSKY_SFC_SW_DWN + so2 + CholesterolF + total_alc
RF1	R_0_~total_alc + OpenUV_max + wind.speed + GDP15_SM + obesity_prev + total_NH3_2015 + urban.population.2018. + total_SO2_2015 + no2 + CLRSKY_SFC_SW_DWN + ALLSKY_SFC_SW_DWN + CholesterolF + so2
RF2	R_0_~total_alc + OpenUV_max + wind.speed + GDP15_SM + obesity_prev + total_NH3_2015 + total_SO2_2015 + Under.5.mort..2018. + no2 + CholesterolF
Union	R_0_~total_alc + OpenUV_max + wind.speed + GDP15_SM + obesity_prev + total_NH3_2015 + urban.population.2018. + total_SO2_2015 + no2 + CLRSKY_SFC_SW_DWN + ALLSKY_SFC_SW_DWN + CholesterolF + so2 + Under.5.mort..2018. + T2MWET + T2M_MIN + Weight + NMVOC_pc + o3 + PPP.per.capita + fast_glucF

**Table 2:** sMSE for all combinations of algorithms and predictor subsets.

Predictor Set	SAIC		Lasso1		Lasso2		Random Forest 1		Random Forest 2		Union	
Dataset Segment	Train	Test	Train	Test	Train	Test	Train	Test	Train	Test	Train	Test
OLS Regression	0.59	1.05	0.79	1.44	0.79	1.42	0.73	1.15	0.79	1.21	0.51	1.08
Robust Regression	0.59	1.05	0.79	1.47	0.79	1.47	0.73	1.23	0.80	1.16	0.52	0.96
PC Regression	0.59	1.05	0.80	1.33	0.80	1.33	0.84	1.04	1.02	1.23	0.95	1.13
Ridge Regression	0.59	0.98	0.79	1.32	0.80	1.25	0.87	1.02	0.94	1.10	0.72	0.78
Lasso Regression	0.59	1.05	0.79	1.44	0.79	1.40	0.85	1.09	1.21	1.19	0.59	0.86
ElasticNet Regression	0.59	0.97	0.79	1.39	0.79	1.30	0.86	1.02	0.92	1.12	0.70	0.78
Random Forest Regression	0.16	0.70	0.17	0.74	0.26	0.88	0.35	0.91	0.14	0.73	0.26	0.83
Support Vector Regression	0.42	0.98	0.52	1.25	0.47	1.17	0.18	0.92	0.34	0.88	0.25	0.89
XGBoost Regression	0.33	0.83	0.50	0.74	0.48	0.82	0.25	0.84	0.23	1.02	0.24	0.77

## Data Availability

All data gathered for this study and the R 4.0, Python 3.9 and MATLAB R2023b (MathWorks) scripts used in the analyses are publicly available at https://github.com/OgnjenMilicevic/covid19ml.

## Supplementary Materials

The following supporting information can be downloaded at: https://www.mdpi.com/article/10.3390/pathogens15090879/s1, Table S1: Complete inventory of the 106 candidate predictor variables evaluated before predictor-coverage screening, together with five auxiliary variables retained for documentation but not used as predictors; Table S2: Tunable parameters obtained in the validation stage for all tested algorithms, shown for each of the six predictor subsets, together with definitions of the reported hyperparameters; Table S3: Absolute standardized coefficients from a post-selection ordinary least-squares refit using the 21-variable Union predictor set.
