# Supplementary material for: Inferring Seasonal Modulation of Early SARS-CoV-2 Transmissibility from Cross-Country Environmental and Population-Level Predictors
Source: Pathogens. 2026 Aug 22;15(9):879. doi: 10.3390/pathogens15090879 (PMC13610035; doi:10.3390/pathogens15090879)
Supplement: Supplementary file 1 [file pathogens-15-00879-s001.zip › pathogens-4459530-supplementary.pdf]

Supplementary material

Table S1: Complete inventory of the 106 candidate predictor variables evaluated before predictor-coverage screening, together with five auxiliary variables retained for documentation (R_0, c, start, end, and Country.Code). Predictor variables are organized into five thematic domains, with auxiliary variables listed separately. Variables with at least 100 missing values across the 118 countries were excluded, leaving 96 candidate predictors.

#	Variable name	Description	Source	Missing
**1. Meteorological and Ultraviolet Conditions**				
1	T2M	Mean air temperature at 2 m (°C)	NASA POWER	0 (0.0%)
2	T2M_MAX	Maximum air temperature at 2 m (°C)	NASA POWER	0 (0.0%)
3	T2M_MIN	Minimum air temperature at 2 m (°C)	NASA POWER	0 (0.0%)
4	T10M	Mean air temperature at 10 m (°C)	NASA POWER	0 (0.0%)
5	T10M_MAX	Maximum air temperature at 10 m (°C)	NASA POWER	0 (0.0%)
6	T10M_MIN	Minimum air temperature at 10 m (°C)	NASA POWER	0 (0.0%)
7	TS	Temperature of the ground surface—skin temperature (°C)	NASA POWER	0 (0.0%)
8	T2MWET	Wet Bulb Temperature at 2 m (°C)	NASA POWER	0 (0.0%)
9	RH2M	Relative humidity at 2 m (%)	NASA POWER	0 (0.0%)
10	QV2M	Specific humidity at 2 m (g/kg)	NASA POWER	0 (0.0%)
11	T2MDEW	Dew point temperature at 2 m (°C)	NASA POWER	0 (0.0%)
12	WS2M	Wind speed at 2 m (m/s)	NASA POWER	0 (0.0%)
13	WS10M	Wind speed at 10 m (m/s)	NASA POWER	0 (0.0%)
14	PRECTOT	Total precipitation (mm/day)	NASA POWER	0 (0.0%)
15	TQV	Total column water vapor (kg/m²)	NASA POWER	0 (0.0%)
16	ALLSKY_SFC_SW_DWN	All-Sky Surface Shortwave Downward Irradiance (W/m²)	NASA POWER	0 (0.0%)
17	CLRSKY_SFC_SW_DWN	Clear-Sky Surface Shortwave Downward Irradiance (W/m²)	NASA POWER	3 (2.5%)
18	ALLSKY_SFC_LW_DWN	All-Sky Surface Longwave Downward Irradiance (W/m²)	NASA POWER	0 (0.0%)
19	OpenUV_max	Maximum daily UV index (population weighted)	OpenUV API	0 (0.0%)
20	temperature	WAQI temperature (directly measured in urban centers, °C)	WAQI	53 (44.9%)
21	uvi	UV index (WAQI)	WAQI	118 (100.0%)
22	dew	Dew temperature (WAQI, °C)	WAQI	60 (50.8%)
23	wind.speed	Wind speed — WAQI (m/s)	WAQI	54 (45.8%)
24	wind.gust	Wind gust — WAQI (m/s)	WAQI	64 (54.2%)
25	humidity	Relative humidity — WAQI (%)	WAQI	53 (44.9%)

#	Variable name	Description	Source	Missing
26	pressure	Atmospheric pressure— WAQI (hPa)	WAQI	53 (44.9%)
27	wind.speed.1	Wind speed (alternative source, m/s)	WAQI	105 (89.0%)
28	wind.gust.1	Wind gust (alternative source, m/s)	WAQI	115 (97.5%)
29	precipitation	Precipitation — WAQI (mm)	WAQI	111 (94.1%)
30	wd	Wind direction — WAQI (°)	WAQI	114 (96.6%)
**2. Sociodemographic, Economic, and Urban-Structure Characteristics**				
1	BUCAP15	Built-up urban area per capita 2015 (m²/res.)	GHS-UCDB R2019A (JRC)	4 (3.4%)
2	GDP15_SM	Gross domestic product at city level 2015 (USD)	GHS-UCDB R2019A (JRC)	4 (3.4%)
3	density	Population density (res./km²)	GHS-UCDB / World Bank	0 (0.0%)
4	urban.population.2018.	Percent urban population, 2018 (%)	World Bank	0 (0.0%)
5	Median.age..CIA.	Population age median (years)	CIA World Factbook	1 (0.8%)
6	over.65....	Percent population aged over 65 (%)	World Bank	4 (3.4%)
7	PPP.per.capita	Purchase power parity per capita (intl. USD)	World Bank	5 (4.2%)
8	HDI...OWID.	Human Development Index	OWID / UNDP	4 (3.4%)
9	life.expectancy.born.1990.	Life expectancy (persons born in 1990.)	World Bank	3 (2.5%)
10	infant.mort..2018.	Infant mortality rate per 1000 liveborn, 2018	World Bank	3 (2.5%)
11	Under.5.mort..2018.	Child mortality aged under 5 per 1000 liveborn, 2018	World Bank	3 (2.5%)
12	prc.rodj.u.inostranstvu.WB.2015.	Percent population born abroad, 2015 (%)	World Bank	0 (0.0%)
13	immigrants.emigrants.2017.	Net migration (immigrants − emigrants), 2017	World Bank	4 (3.4%)
14	n.nastanjenih.izbeglica.2018.	Number of resident refugees, 2018	UNHCR	7 (5.9%)
15	Smokers.2016	Smoking prevalence, both genders, modelled from trend, 2016 (%)	WHO GHO	19 (16.1%)
16	Population_2015	Total population, 2015	World Bank	0 (0.0%)
17	Weight	Ratio of urban to total population; used in population exposure scaling for variables measured in urban setting (e.g. WAQI); considered a proxy for urban concentration or confidence score for environmental data quality	Calculated from World Bank and GHS-UCDB R2019A	0 (0.0%)

#	Variable name	Description	Source	Missing
**3. Population-Health Indicators**				
1	Raised.BP	Prevalence of increased blood pressure, adults (%)	WHO GHO (BP_04)	5 (4.2%)
2	CholesterolM	Mean cholesterol — men (mmol/L)	WHO GHO (CHOL_03)	5 (4.2%)
3	CholesterolF	Mean cholesterol — women (mmol/L)	WHO GHO (CHOL_03)	5 (4.2%)
4	CHEpGDP	Current health expenditures as % BDP-a	WHO GHED	6 (5.1%)
5	doc10K	Number of physicians per 10,000 residents	WHO HWF	4 (3.4%)
6	pharm10K	Number of pharmacists per 10,000 residents	WHO HWF	10 (8.5%)
7	tobaccoMFS	Smoking prevalence from interviews, both genders standardized (%)	WHO GHO	22 (18.6%)
8	suicideMF2016	Suicide rate per 100,000 — both genders, 2016	WHO GHO	6 (5.1%)
9	obesity_prev	Obesity prevalence in adults (%)	WHO GHO (NCD_BMI_30A)	5 (4.2%)
10	meanBMI	Average Body Mass Index (BMI)	WHO GHO (NCD_BMI_MEAN)	5 (4.2%)
11	fast_glucM	Average fasting glucose — men (mmol/L)	WHO GHO (NCD_GLUC_01)	5 (4.2%)
12	fast_glucF	Average fasting glucose — women (mmol/L)	WHO GHO (NCD_GLUC_01)	5 (4.2%)
13	inactive	Prevalence of physical inactivity (%)	WHO GHO (NCD_PAC/PAA)	21 (17.8%)
14	prob_death	Probability of premature death from nonchronic disease (30–70 god.), %	WHO GHO (NCDMORT3070)	6 (5.1%)
15	mort_WASH	Mortality contributed to bad WASH conditions (per 100,000)	WHO GHO (SDGWSHBOD)	6 (5.1%)
16	total_alc	Total alcohol consumption per capita (liters of pure alcohol)	WHO GHO (WHOSIS_000011)	5 (4.2%)
17	BCG1	BCG vaccination coverage (%)	WHO GHO (WHS4_543)	33 (28.0%)
18	hosp_beds10K	Number of hospital beds per 10,000 residents	WHO GHO (WHS6_102)	6 (5.1%)
19	basic_washU	Access to basic hygiene, urban population (%)	WHO GHO (WSH_HYGIENE_BASIC)	83 (70.3%)
20	open_defT2017	Prevalenca of open defecation — total, 2017 (%)	WHO GHO	10 (8.5%)
21	bas_sanU2017	Access to basic sanitation, urban population, 2017 (%)	WHO GHO	17 (14.4%)

#	Variable name	Description	Source	Missing
22	bas_watU2017	Access to safe water, urban population, 2017 (%)	WHO GHO	16 (13.6%)
23	CVD_canc_diab_CRD.2016.	Cardiovascular, cancer, diabetes and chronic respiratory disease mortality, 2016 (per 100,000)	WHO GHO	5 (4.2%)
24	Share.adults.obese.OWID.	Percent overweight adults (%)	OWID	4 (3.4%)
25	avg.BMI...OWID.	Average BMI	OWID	4 (3.4%)
**4. Air-Pollution Indicators**				
1	pollution.2.5.2017.	Concentration $PM_{2.5}$, 2017 (μg/m³)	WAQI / satellite data	4 (3.4%)
2	pollution.2.5.2013.2017.avg	Average $PM_{2.5}$ concentration 2013–2017 (μg/m³)	WAQI / satellite data	4 (3.4%)
3	PM2.5_2019	$PM_{2.5}$ concentration, 2019 (μg/m³)	IQAir World Air Quality Report	44 (37.3%)
4	pm25	$PM_{2.5}$ (WAQI direct measurement, μg/m³)	WAQI	59 (50.0%)
5	pm10	PM_{10} (WAQI direct measurement, μg/m³)	WAQI	73 (61.9%)
6	pm1	PM_1 (WAQI direct measurement, μg/m³)	WAQI	117 (99.2%)
7	no2	NO_2 (WAQI, μg/m³)	WAQI	75 (63.6%)
8	o3	O_3 — ozone (WAQI, μg/m³)	WAQI	79 (66.9%)
9	so2	SO_2 (WAQI, μg/m³)	WAQI	78 (66.1%)
10	co	CO — carbon-monoxide (WAQI, μg/m³)	WAQI	84 (71.2%)
11	aqi	Air Quality Index — AQI (WAQI)	WAQI	117 (99.2%)
12	pol	Dominant pollutant (categorical)	WAQI	117 (99.2%)
13	neph	Nephelometry — optic indicator of air turbidity	WAQI	117 (99.2%)
14	mepaqi	MEP AQI index (Chinese scale)	WAQI	117 (99.2%)
15	total_NOx_2015	Total emission NO_x, 2015 (kt/yr.)	EDGAR v5.0	4 (3.4%)
16	NOx_pc	Emissions NO_x per capita, 2015	EDGAR v5.0	4 (3.4%)
17	total_BC_2015	Total emission of black carbon (BC), 2015 (kt/yr.)	EDGAR v5.0	4 (3.4%)
18	BC_pc	Emissions BC per capita, 2015	EDGAR v5.0	4 (3.4%)
19	total_CO_2015	Total emission CO, 2015 (kt/yr.)	EDGAR v5.0	4 (3.4%)
20	CO_pc	Emission CO per capita, 2015	EDGAR v5.0	4 (3.4%)
21	total_NH3_2015	Total emission NH_3, 2015 (kt/yr.)	EDGAR v5.0	4 (3.4%)
22	NH3_pc	Emission NH_3 per capita, 2015	EDGAR v5.0	4 (3.4%)
23	total_NMVOC_2015	Total emission NMVOC, 2015 (kt/yr.)	EDGAR v5.0	4 (3.4%)
24	NMVOC_pc	Emission NMVOC per capita, 2015	EDGAR v5.0	4 (3.4%)

#	Variable name	Description	Source	Missing
25	total_OC_2015	Total emission organic carbon (OC), 2015 (kt)	EDGAR v5.0	4 (3.4%)
26	OC_pc	Emission OC per capita, 2015	EDGAR v5.0	4 (3.4%)
27	total_PM10_2015	Total emission PM_{10}, 2015 (kt/yr.)	EDGAR v5.0	4 (3.4%)
28	PM10_pc	Emission PM_{10} per capita, 2015	EDGAR v5.0	4 (3.4%)
29	total_SO2_2015	Total emission SO_2, 2015 (kt/yr.)	EDGAR v5.0	4 (3.4%)
30	SO2_pc	Emission SO_2 per capita, 2015	EDGAR v5.0	4 (3.4%)
31	total_PM2.5_2015	Total emission $PM_{2.5}$, 2015 (kt/yr.)	EDGAR v5.0	4 (3.4%)
32	PM2.5_pc	Emission $PM_{2.5}$ per capita, 2015	EDGAR v5.0	4 (3.4%)
**5. Social Connectedness**				
1	Flow.Betweenness. Centrality	Flow betweenness centrality of the country in the SCI network	Facebook SCI (Bailey et al., 2018)	9 (7.6%)
2	Weighted.Vertex.Degree	Weighted vertex degree in the SCI network	Facebook SCI (Bailey et al., 2018)	9 (7.6%)
**Auxiliary Variables—Not Used as Predictors**				
1	R0	Estimated basic reproduction number (dependent variable)	Estimated from epidemiological data	0 (0.0%)
2	c	Exponential growth rate λ^+ (dan^{-1})	Estimated from epidemiological data	0 (0.0%)
3	start	Date of exponential phase beginning for the given country	Worldometer / Our World in Data	0 (0.0%)
4	end	Date of exponential phase end for the given country	Worldometer / Our World in Data	0 (0.0%)
5	Country.Code	ISO 3166-1 alpha-3 country code	ISO 3166	0 (0.0%)

Table S2: Tunable parameters obtained in the validation stage for all the tested algorithms, shown for each of the 6 predictor subsets

Method	PCR	Ridge	Lasso	Elastic Net		Random Forest			Support Vector		XGBoost						
set	N	λ	λ	λ	α	mtry	mns	spr	ε	C	nrounds	eta	mxd	γ	cls	mcw	sbs
saic	5	0.158	0.013	0.061	0.1	5	4	extratrees	0.35	2	300	0.01	2	0.05	0.8	1	0.5
lasso1	2	0.501	1e-05	0.265	0.1	1	3	extratrees	0.49	1	250	0.01	2	0.1	0.8	1	0.75
lasso2	2	0.501	0.01	0.299	0.1	5	10	extratrees	0.39	0.5	400	0.005	2	0.5	0.6	1	1
rf1	2	0.794	0.126	0.343	0.1	3	9	extratrees	0.01	2	750	0.005	2	0.9	0.4	2	0.75
rf2	2	0.501	0.040	0.284	0.1	3	9	extratrees	0.31	1	600	0.005	2	0.7	0.4	1	0.75
uni	3	0.631	0.032	0.218	0.1	4	6	extratrees	0.11	1	750	0.005	2	0.7	0.4	2	0.5

Explanation of hyperparameters and values:

- Principal Component Regression (PCR)
  - **N**: Number of principal components retained in the model to capture the maximum variance while reducing feature dimensionality.

- Regularized Linear Models
  - λ_Ridge: The penalty parameter lambda for Ridge regression, dictating the strength of the L2 regularization (shrinkage).
  - λ_Lasso: The penalty parameter lambda for Lasso regression, dictating the strength of the L1 regularization (feature selection).
  - λ_EN: The overall penalty parameter lambda applied in Elastic Net regression.
  - α: The mixing parameter alpha for Elastic Net, which balances the ratio between the L1 (Lasso) and L2 (Ridge) penalties.

- Random Forest (RF)
  - **mtry**: The number of predictor variables randomly sampled as candidates at each node split.
  - **mns**: Minimum node size; the minimum number of data points allowed in a terminal node (leaf) before stopping further splits.
  - **spr**: The splitting rule used to partition the data (e.g., "extratrees" stands for Extremely Randomized Trees, which introduces additional stochasticity by randomly selecting split thresholds).

- Support Vector Regression (SVR)
  - ε: The epsilon parameter, defining a margin of tolerance (the epsilon-tube) within which errors are not penalized.
  - **C**: The cost parameter which determines the penalty applied to predictions that fall outside the defined epsilon margin.

- Extreme Gradient Boosting (XGBoost)
  - **nrounds**: The total number of sequential boosting iterations (i.e., the number of trees built).
  - **eta**: The learning rate which shrinks the feature weights after each boosting step to make the model more robust and prevent overfitting.
  - **mxd**: Maximum tree depth, setting the limit on how many splits a single tree can have from root to leaf.
  - γ: Gamma, representing the minimum reduction in loss required to justify creating a new split in a tree node.
  - **cls**: Column subsampling ratio (`colsample_bytree`), determining the fraction of features randomly supplied to each individual tree.
  - **mcw**: Minimum child weight, the minimum sum of instance weights (Hessian) required in a child node to permit a further partition.
  - **sbs**: Subsampling ratio, defining the percentage of the training data randomly sampled prior to growing each tree.

Table S3: Absolute standardized coefficients from an auxiliary post-selection ordinary least-squares refit using the 21-variable Union predictor set. The refit was used as a descriptive coefficient-based summary and was not used for model selection or annual $R_0(t)$ reconstruction. Because correlated predictors share explanatory information, the reported coefficients should not be interpreted as independent variable contributions or causal effects; see Discussion.

Rank	Variable	Absolute standardized coefficient	Domain
1	T2M_MIN	0.435	Meteorological and Ultraviolet Conditions
2	PPP.per.capita	0.295	Sociodemographic, Economic, and Urban-Structure Characteristics
3	obesity_prev	0.286	Population-Health Indicators
4	NMVOC_pc	0.277	Air-Pollution Indicators
5	GDP15_SM	0.247	Sociodemographic, Economic, and Urban-Structure Characteristics
6	T2MWET	0.239	Meteorological and Ultraviolet Conditions
7	Weight	0.221	Sociodemographic, Economic, and Urban-Structure Characteristics
8	total_SO2_2015	0.202	Air-Pollution Indicators
9	fast_glucF	0.188	Population-Health Indicators
10	o3	0.185	Air-Pollution Indicators
11	CLRSKY_SFC_SW_DWN	0.178	Meteorological and Ultraviolet Conditions
12	wind.speed	0.156	Meteorological and Ultraviolet Conditions
13	no2	0.119	Air-Pollution Indicators
14	total_NH3_2015	0.106	Air-Pollution Indicators
15	total_alc	0.105	Population-Health Indicators
16	so2	0.095	Air-Pollution Indicators
17	ALLSKY_SFC_SW_DWN	0.079	Meteorological and Ultraviolet Conditions
18	CholesterolF	0.077	Population-Health Indicators
19	Under.5.mort..2018.	0.054	Sociodemographic, Economic, and Urban-Structure Characteristics
20	OpenUV_max	0.044	Meteorological and Ultraviolet Conditions
21	urban.population.2018.	0.035	Sociodemographic, Economic, and Urban-Structure Characteristics
